# *Apiaceae* Bioferments Obtained by Fermentation with Kombucha as an Important Source of Active Substances for Skin Care

**DOI:** 10.3390/molecules30050983

**Published:** 2025-02-20

**Authors:** Zofia Nizioł-Łukaszewska, Aleksandra Ziemlewska, Martyna Zagórska-Dziok, Agnieszka Mokrzyńska, Magdalena Wójciak, Ireneusz Sowa

**Affiliations:** 1 Department of Technology of Cosmetic and Pharmaceutical Products, Medical College, University of Information Technology and Management in Rzeszow, Sucharskiego 2, 35-225 Rzeszow, Poland; aziemlewska@wsiz.edu.pl (A.Z.); mzagorska@wsiz.edu.pl (M.Z.-D.); amokrzynska@wsiz.edu.pl (A.M.); 2 Department of Analytical Chemistry, Medical University of Lublin, Aleje Raclawickie 1, 20-059 Lublin, Poland; magdalena.wojciak@umlub.pl (M.W.); ireneusz.sowa@umlub.pl (I.S.)

**Keywords:** *Apium graveolens* L., *Daucus carota* L., *Petroselinum crispum* (Mill.) Fuss, kombucha, skin cells, antioxidants, cytotoxicity, anti-inflammatory activity, antibacterial activity

## Abstract

This article attempts to comprehensively assess plants from the *Apiaceae* family, such as *Apium graveolens*, *Daucus carota* or *Petroselinum crispum*, as raw plant materials with potential uses in cosmetic products with anti-inflammatory and antibacterial effects. The work compares the phytochemical profiles and activity of extracts and ferments from the roots of these plants obtained during fermentation using kombucha. The antioxidant properties of the tested extracts, the effect on the intracellular level of free radicals and their cytotoxicity towards skin cells were compared. Their anti-inflammatory and antibacterial properties were also assessed. The ABTS and DPPH tests indicated the highest antioxidant potential of the carrot ferments, achieving a 55.75% and 74.6% reduction of these radicals, respectively. The resazurin and Neutral Red assays indicated that in most cases, sample concentrations not exceeding 2.5% did not cause a cytotoxic effect, and in the case of a 20-day parsley ferment, they could increase viability by over 40%. The disk diffusion method indicated growth inhibition zones of over 20 mm for some bacteria. The minimum inhibitory concentrations for seven different bacterial strains ranged from 200 to 400 µg/mL. Anti-inflammatory properties were determined using the ELISA method, assessing the level of interleukins 1β, 6 and 10. The obtained results indicate a higher amount of phytochemicals, a lack of cytotoxic effect at lower concentrations of the tested samples and significantly stronger antioxidant, antibacterial and anti-inflammatory properties of the ferments compared to the extracts. This effect depends on the concentration and fermentation time used.

## 1. Introduction

Natural plant substances play a key role in skin care, thanks to the content of numerous chemical substances that can be secreted by cells as physiological components or as by-products of metabolism. The content of bioactive substances in plant materials affects many health-promoting properties, such as cytotoxic, anti-inflammatory, immunomodulatory or antibacterial effects. In addition, a very large group of secondary metabolites exhibit strong antioxidant properties [1,2]. These compounds affect the proper maintenance of redox balance by regulating oxidation-reduction processes in cells. Disturbances of the redox balance caused by the excessive production of free radicals contribute to structural and functional changes in the skin, which can ultimately result in cell death [3,4]. In damaged cells, membrane lipids’ peroxidation and the modification of membrane proteins occur, which also contributes to the increased production of pro-inflammatory cytokines and, as a result, to the development of inflammation [3,4,5,6].

Plant materials that exhibit antioxidant properties and strong anti-inflammatory or antibacterial effects include plants belonging to the *Apiaceae* family, such as *Apium graveolens* L. (celery), *Daucus carota* L. (carrota) or *Petroselinum crispum* (Mill.) Fuss (parsley).

In the case of *A. graveolens*, it has been shown that the presence of active substances such as flavonoids (apigenin or luteoin) in the extracts from different parts of plants can modify the inflammatory response, which may be beneficial in the treatment of many inflammatory skin conditions [7]. The presence of active compounds contained in the extract from *A. graveolens* demonstrates the ability to inhibit the activity of pro-inflammatory mediators, such as cytokines IL-1β and IL-6, as well as inflammatory enzymes such as cyclooxygenase-2 (COX-2) [8]. *A. graveolens* extracts demonstrate the ability to inhibit the growth of bacterial strains, both Gram-positive and Gram-negative, by interacting with their cell membranes. Compounds such as terpenoids and flavonoids can react with bacterial cell membranes, leading to their destabilization, which results in the loss of cellular integrity and thus inhibition of pathogen growth. *A. graveolens* extracts from different parts of plants have soothing properties, reducing redness and swelling of the skin [7,8,9].

In addition, *P. crispum* extract has strong antioxidant and anti-inflammatory effects, which may be due to the presence of flavonoids and other phenolic compounds such as quercetin, apigenin, luteolin and rutin. It has also been shown that both root and aerial extracts of this plant can reduce the concentration of pro-inflammatory cytokines, such as TNF-α and IL-6, reducing the production of inflammatory mediators and improving the skin barrier function. *P. crispum* has also the ability to inhibit bacterial enzymes, such as proteases and amylases, which are essential for bacterial survival and multiplication [10,11,12,13,14].

*D. carota*, due to its content of phenolic acids, such as chlorogenic acid, has antioxidant properties and has the ability to inhibit COX-2 enzymes (cyclooxygenase-2), which are responsible for the synthesis of pro-inflammatory prostaglandins [7,14,15]. In addition, the beta-carotene in *D. carota* root and peel extracts plays an important role in reducing the level of reactive oxygen species (ROS) and also has an immunomodulatory effect by reducing the production of pro-inflammatory cytokines, such as IL-1β and IL-6, which makes it effective in relieving the symptoms of skin inflammation, such as redness or swelling [15,16,17,18,19].

Currently, in the cosmetic and pharmaceutical industry, it is very important to search for innovative ingredients whose active substances are characterized by high bioavailability and which we can successfully use in cosmetic products. In the market, we observe not only the demand for products with a high content of raw materials of natural origin but also for sustainable products that will be characterized by high quality, effectiveness of use and a high level of safety [2,4,5]. Particular attention has been paid to the process of fermentation of plant materials using a symbiotic culture of bacteria and yeast (SCOBY) called kombucha. The fermentation of kombucha with the addition of plant materials, occurring as a result of the action of the symbiotic culture, allows for obtaining bioactive metabolites that have anti-inflammatory, irritation-soothing and antibacterial properties [19,20]. Additionally, the polyphenols contained in kombucha have antioxidant effects, protecting skin cells from damage caused by free radicals. The microorganisms contained in kombucha, such as lactic acid bacteria and yeast, may also have a beneficial effect on the skin microbiome by reducing inflammation and skin problems such as acne and eczema [21,22]. In addition, the active substances contained in plant ferments can contribute to reducing the irritating potential of cosmetic products. They can delay the breakdown of unsaturated lipids in the extracellular matrix and thus protect the skin from damage to the epidermal barrier, ultimately reducing the potential risk of skin irritation and limiting anti-inflammatory processes [3,4,5,6,19,20,21].

In this study, an attempt was made to assess the quality of the extracts and ferments obtained from the *Apiaceae* family, such as *A. graveolens*, *Daucus carota* or *Petroselinum crispum*. For this purpose, analyses were carried out to determine the content of biologically active compounds in extracts and ferments. The level of cytotoxicity was assessed, antioxidant properties were examined and the level of ROS in skin cells exposed to kombucha extracts and ferments was evaluated. The experiments were conducted on two human cell lines: keratinocytes (HaCaTs) and fibroblasts (BJ). In addition, the expression of pro- and anti-inflammatory cytokines was determined and the antibacterial properties of the obtained extracts and ferments were assessed.

## 2. Results and Discussion

### 2.1. HPLC Analysis

**Figure 1:**
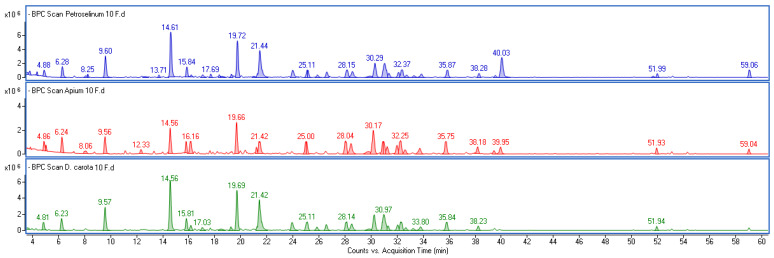
Base peak chromatogram of 10-day fermented extracts from *P. crispum* (blue line), *A. graveolens* (red line) and *D. carota* (green line). MS data extracted from particular peaks and the names of identified components are given in Table 1.

Base peak chromatograms (BPCs) and ultraviolet–visible (UV-Vis) chromatograms of water extracts from the roots of parsley (*P. crispum*), carrot (*D. carota*) and celery (*A. graveolens*) showed only a few small peaks (Figure S1a), with MS data provided in Tables S1–S3. Among these, apigenin-7-apio-glucoside (Apiin) (*m/z*-H in the range of 563.138–563.143, estimated formula C_26_H_28_O_14_) with a fragment ion typical for the aglycone (*m/z* = 269) and the characteristic UV-Vis spectrum (Figure S1b), was identified as the predominant polyphenolic constituent in the water extracts of parsley and celery roots, which is in agreement with literature data [23]. The concentrations of Apiin were 3.4 ± 0.2 and 0.18 ± 0.2 µg/mL, respectively. Apigenin and apigenin 7-glucoside were also found in both extracts. Furthermore, a few components belonging to the fatty acid class were identified, including trihydroxyoctadecadienoic acid, trihydroxyoctadecenoic acid and octadecenedioic acid. During sample processing, the phenolic profile of fermented extracts changed significantly compared to non-fermented extracts. Interestingly, the qualitative profile of the ferment from all investigated roots was very similar (Figure 1).

The predominant constituents of fermented extracts are compounds with common ions at *m/z*-H = 169 and *m/z*-H = 125, resulting from decarboxylation, and were assigned as gallic acid derivatives. The most abundant were identified as free gallic acid with *m/z*-H = 169.015 (estimated formula: C_7_H_6_O_5_) and galloylquinic acid isomers with *m/z*-H = 343.068 (estimated formula:C_14_H_16_O_10_), which show an additional fragment ion at *m/z*-H = 191, indicating the presence of quinic acid in the molecule’s structure. The second most abundant group of components possesses a common fragment ion at *m/z*-H = 289, and they were tentatively identified as (epi)catechin and (epi)catechin derivatives. Among them, free epicatechin with *m/z*-H = 289.072 (estimated formula: C_15_H_14_O_6_) and epigallocatechin gallates with a parent ion at *m/z*-H = 457.076 (estimated formula: C_22_H_18_O_11_), along with fragment ions corresponding to the gallic acid and its decarboxylation product (*m/z* = 169 and *m/z*-H = 125, respectively), were found in the highest concentration.

Flavonoid compounds were also detected. Kaempferol derivatives, including rutinoside (*m/z*-H = 593.151; estimated formula: C_27_H_30_O_15_), glucoside (*m/z*-H = 447.092; estimated formula: C_21_H_20_O_11_) and unidentified compounds with *m/z*-H = 593.153 and *m*/*z*-H = 755.203 showed a common ion at *m/z*-H = 285. Quercetin derivatives, with a common ion at *m/z*-H = 300, were identified as rutinoside (*m/z*-H = 609.147; estimated formula: C_27_H_30_O_16_), galactoside and glucoside (both *m/z*-H = 463.087; estimated formula: C_21_H_20_O_12_). Derivatives of apigenin (with a common fragment ion at *m/z*-H = 269) were represented by 7-apioglucoside (*m/z*-H = 563.143; estimated formula: C_26_H_28_O_14_) and 7-glucoside (*m/z*-H = 431.097; estimated formula: C_21_H_20_O_10_).

Furthermore, chlorogenic acids (caffeoylquinic isomers) were identified based on *m/z*-H = 353.089 (estimated formula: C_16_H_18_O_9_) and characteristic fragment ions, including *m/z*-H = 179, corresponding to caffeic acid, and *m/z*-H = 191, corresponding to quinic acid.

Identification was confirmed by comparison with standards when available, or compounds were tentatively identified based on mass data and UV-Vis spectra. Formulas were generated using MassHunter software (ver. 3.3.2 SP2 build 3.3.2.1037), with the maximal difference between measured and theoretical mass set at 5 ppm. Detailed mass data are included in Table 1.

The quantity of particular metabolites from the gallic acid and catechin derivative classes differed depending on the plant material used for processing; however, chlorogenic acids and flavonoids were almost at the same level. Fermentation time had a minor effect on the quantitative profile, with the exception of gallic acid in the parsley extract, which increased twofold. Detailed results of the quantification of the main metabolites are shown in Table 2.

### 2.2. Assessment of Antioxidant Activity

#### 2.2.1. ABTS and DPPH Radical Scavenging

Extracts and ferments of roots from *A. graveolens*, *D. carota* and *P. crispum* were subjected to the evaluation of their antioxidant activity. For this purpose, two spectrophotometric methods were used: ABTS and DPPH. Both methods are based on the reaction with free radicals and the assessment of the antioxidants’ ability to neutralize them, which makes them effective tools in the analysis of the antioxidant potential of ingredients used in skin care products. The ABTS method is based on the generation of the ABTS radical, which is reduced in the presence of an antioxidant, leading to a decrease in color intensity. In turn, the DPPH method uses the DPPH radical, characterized by a purple color, which disappears in the presence of antioxidants. Both methods are widely used in studies on the antioxidant activity of cosmetics, plant extracts and raw materials used in skin care products.

**Figure 2:**
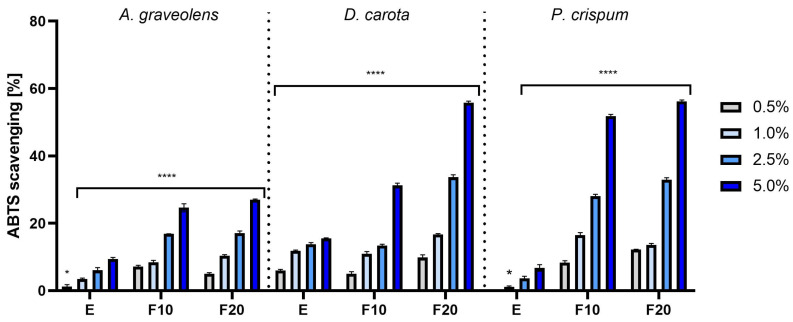
The ability of *A. graveolens*, *D. carota* and *P. crispum* root extracts (E) and ferments (F10 and F20) to scavenge ABTS free radicals at concentrations of 0.5%, 1.0%, 2.5% and 5.0%. Data are presented as mean ± SD from three independent experiments, with each sample tested in triplicate. **** *p* < 0.0001, * *p* < 0.05.

**Figure 3:**
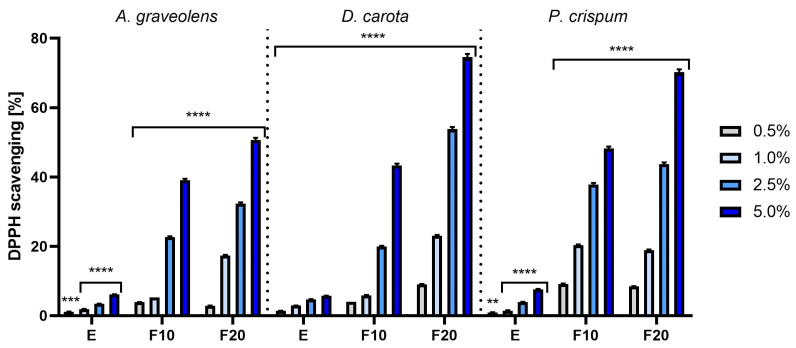
The ability of *A. graveolens*, *D. carota* and *P. crispum* roots extracts (E) and ferments (F10 and F20) to scavenge DPPH free radicals at concentrations of 0.5%, 1.0%, 2.5% and 5.0%. Data are presented as mean ± SD from three independent experiments, with each sample tested in triplicate. **** *p* < 0.0001, *** *p* = 0.0001, ** *p* = 0.002.

The analysis of antioxidant properties of extracts and ferments covered the concentration range from 0.5% to 5.0% and the results are presented as the percentage of free radical scavenging capacity (Figure 2 and Figure 3). The obtained results significantly showed that extracts fermented with tea fungus kombucha were characterized by stronger antioxidant activity compared to unfermented extracts. A clear relationship between the concentration and free radical scavenging efficiency was also observed—higher concentrations of extracts and ferments resulted in a greater ROS scavenging capacity in both applied tests. In the ABTS and DPPH methods, F20 showed the highest antioxidant activity, reaching 55.75% ± 0.49 and 74.6% ± 0.90 for carrot at a 5.0% concentration, respectively (Figure 2 and Figure 3).

The analyses conducted using ABTS and DPPH methods showed that the fermented extracts were characterized by higher antioxidant activity compared to the unfermented extracts. This may suggest that the fermentation process increases the bioavailability of antioxidant compounds, which makes these ingredients particularly valuable in cosmetic formulations aimed at protecting the skin against oxidative stress [24]. Additionally, the chromatographic analysis allowed the identification of bioavailable compounds contained in extracts and ferments from celery, carrot and parsley roots. The analyses showed that the ferments have a higher content of active ingredients than the unfermented extracts, which could have a significant impact on their higher effectiveness in neutralizing free radicals (Table 2 and Tables S1–S3). The chromatographic analysis showed the presence of compounds such as gallic acid, catechin, epicatechin and chlorogenic acid (Table 2). These compounds have proven antioxidant properties [25,26,27,28]. 

#### 2.2.2. Intracellular ROS Levels in Skin Cells

**Figure 4:**
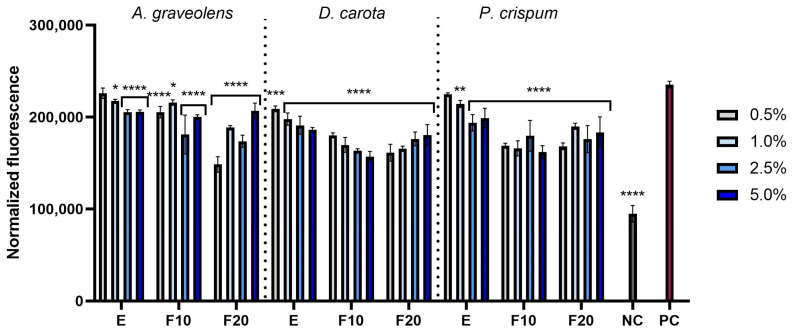
The effect of *A. graveolens, D. carota* and *P. crispum* root extracts (E) and ferments (F10 and F20) at the concentrations of 0.5%, 1.0%, 2.5% and 5.0% on the intracellular level of reactive oxygen species in fibroblasts (HDFs). Data are presented as mean ± SD from three independent experiments, with each sample tested in triplicate. **** *p* < 0.0001, *** *p* < 0.001, ** *p* = 0.0057, * *p* < 0.05.

**Figure 5:**
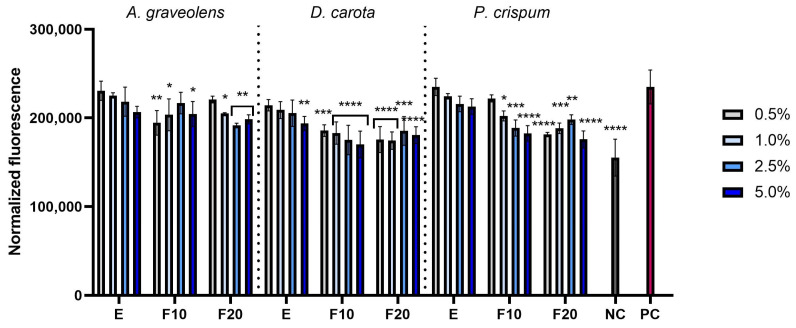
The effect of *A. graveolens, D. carota* and *P. crispum* root extracts (E) and ferments (F10 and F20) at the concentrations of 0.5%, 1.0%, 2.5% and 5.0% on the intracellular level of reactive oxygen species in keratinocytes (HaCaTs). Data are presented as mean ± SD from three independent experiments, with each sample tested in triplicate. **** *p* < 0.0001, *** *p* < 0.001, ** *p* < 0.01, * *p* < 0.05.

This study assessed the ability of celery root, carrot and parsley extracts and ferments to reduce intracellular levels of reactive oxygen species in skin cells induced by hydrogen peroxide. The fluorescent dye H_2_DCFDA was used in the analyses; it is oxidized in the presence of reactive oxygen species, transforming into fluorescent 2′,7′-dichlorofluorescein (DCF). The analyses were performed on two cell lines: fibroblasts and keratinocytes. The results are presented as fluorescence levels. Two control groups were used to determine the effectiveness of the tested substances. The positive control (PC) included cells treated with hydrogen peroxide (H_2_O_2_), but not exposed to extracts or ferments, which was intended to induce oxidative stress. The negative control (NC) consisted of cells that were not treated with either H_2_O_2_ or the tested substances, representing the natural level of ROS. The analyses performed showed that both extracts and ferments showed the ability to reduce oxidative stress (Figure 4 and Figure 5), which was confirmed in the case of both fibroblasts (HDFs) and keratinocytes (HaCaTs). The greatest potential to reduce the level of reactive oxygen species was shown by carrot for both cell lines. In the case of *A. graveolens*, the greatest ability to reduce the level of reactive oxygen species was observed for F20 for both HDFs and HaCaTs. In contrast, for *D. carota* and *P. crispum*, the greatest potential to reduce oxidative stress was shown by F10 for both cell lines.

Plants play an important role as a source of biologically active natural products of cosmetic and dermatological importance. Thanks to the presence of antioxidants such as polyphenols, flavonoids and carotenoids, they can effectively protect the skin from the harmful effects of external factors [25,29]. One of the key threats to skin health is oxidative stress, resulting from the excessive production of reactive oxygen species (ROS). The accumulation of ROS contributes to the oxidation of lipids of cell membranes, damage to structural proteins of the skin (e.g., collagen and elastin) and DNA mutations. The effect is a loss of firmness, the formation of wrinkles, discoloration and skin hypersensitivity [30]. It has been proven that active substances with antioxidant properties, which are contained in plant extracts and ferments, can contribute to the inhibition or quenching of free radical reactions [31,32].

Free radicals and other reactive oxygen species are naturally produced during metabolic processes. In properly functioning cells, their levels are controlled by the antioxidant system, which maintains redox balance. However, in conditions of excessive oxidative stress, caused by UV radiation or environmental pollution, natural defense mechanisms may prove insufficient, resulting in damage to cellular structures.

This study evaluated the ability of extracts and ferments from celery (*Apium graveolens*), carrot (*Daucus carota*) and parsley (*Petroselinum crispum*) to reduce intracellular levels of reactive oxygen species in skin cells exposed to hydrogen peroxide (H_2_O_2_). The results showed that both extracts and ferments had the ability to reduce ROS levels, but the efficiency of ferments was significantly higher than that of extracts. This may be due to the higher content of bioactive compounds. Chromatographic analysis confirmed the presence of numerous phenolic compounds, such as gallic acid, catechins, rutoside and chlorogenic acid (Table 2). All these compounds are known for their strong antioxidant properties, which consist of the ability to neutralize free radicals by donating an electron or a hydrogen atom [33]. Chlorogenic acid may play an important role, as it has the ability to inhibit lipid peroxidation [34]. Gallic acid is capable of removing superoxide anions, among others, and also has the ability to improve the antioxidant status of the body by restoring the activity of superoxide dismutase or catalase [26,35]. Furthermore, the polyphenols present in ferments may have better bioavailability and stability compared to those contained in extracts, which may additionally affect their effectiveness in reducing ROS [27].

Carrot ferments showed particularly high antioxidant potential, which may be due to the high content of carotenoids, which are strong free radical scavengers [36,37]. In the case of celery, the highest effectiveness was demonstrated by the ferment marked as F20, while for parsley and carrots the best results were obtained for the F10 ferment. These results suggest that the fermentation process may increase the availability and activity of antioxidants by degrading larger phenolic complexes into more active forms [38].

### 2.3. Cytotoxicity Assessment

The Alamar Blue (AB) and Neutral Red (NR) tests are commonly used to assess the effect of extracts and ferments with potential cosmetic applications on cell viability and metabolic activity. The AB assay allows for the assessment of the viability of the tested cells by determining the activity of the mitochondrial respiratory chain, which allows for the assessment of cell viability, proliferation and mitochondrial respiratory activity. The NR test, on the other hand, assesses the ability of living cells to absorb and retain Neutral Red dye in lysosomes, which reflects the integrity of cell membranes and cell viability [39,40]. The conducted studies assessed the effect of the tested extracts and ferments on both epidermal and dermal cells, and analyses were performed on keratinocytes (HaCaTs) and fibroblasts (HDFs). In each series, three plan varieties were used: *A. graveolens*, *D. carota* and *P. crispum*. For each of them, pure extract and ferments after 10 and 20 days were used, each in the four concentrations of 0.5%, 1.0%, 2.5% and 5.0%.

**Figure 6:**
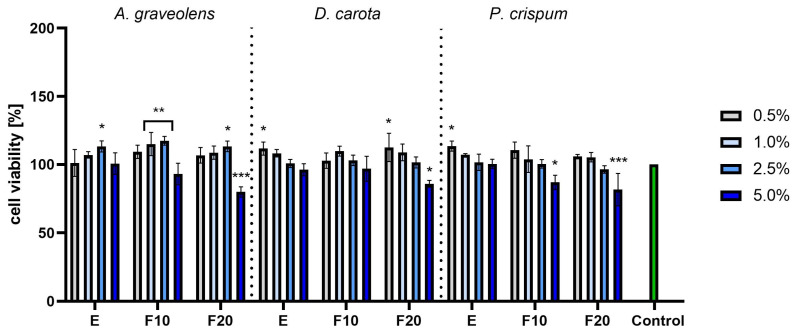
The reduction in resazurin after 24 h exposure to *A. graveolens, D. carota* and *P. crispum* extracts in cultured fibroblasts (HDFs). Data are the means ± SD of three independent experiments in which each sample was tested in three replicates. *** *p* < 0.001, ** *p* < 0.01, * *p* < 0.05.

Studies conducted on fibroblasts (HDFs) using the AB assay showed that the optimal concentration depends on the plant and fermentation level. For *A. graveolens*, the F10 ferments in concentrations of 1.0% and 2.5% gave the highest results, while values observed for the pure extract and F20 ferment in the concentration of 2.5% were also high, but a bit lower than the aforementioned F10 ferment. Slightly lower numbers, but still significantly higher compared to the control, were observed for the F20 ferment from *D. carota*, together with the pure extract of it, both in the lowest concentrations of 0.5%. For *P. crispum*, the only case when values exceeded the control significantly was for the pure extract in the lowest concentration of 0.5%. Except for these few discussed cases, the majority of the measurements remained on a level comparable to the control. On the other hand, in each experiment, the values obtained for the highest concentration of 5.0% remained below the control level. Additionally, it can be noticed that for the discussed concentration of 5.0%, the longer the fermentation time, the more noticeable the drop in cell viability, resulting in statistically significant drop for the combination of F20 and 5.0% for each species. This suggests a cytotoxic effect when a long fermentation time is combined with a high concentration (Figure 6).

**Figure 7:**
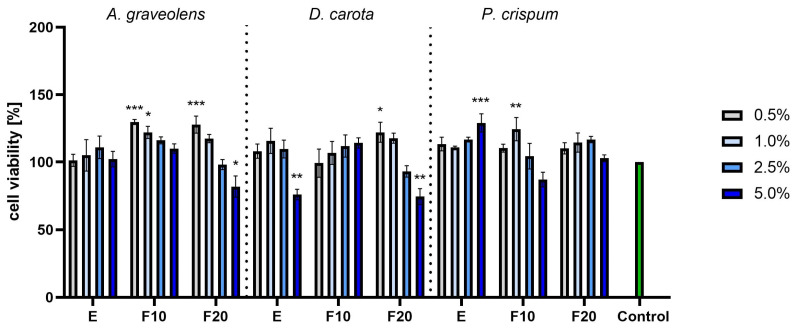
The reduction in resazurin after 24 h exposure to *A. graveolens, D. carota* and *P. crispum* extracts in cultured keratinocytes (HaCaTs). Data are the means ± SD of three independent experiments in which each sample was tested in three replicates. *** *p* < 0.001, ** *p* < 0.01, * *p* < 0.05.

The next series of experiments were conducted for the HaCaT line with the AB test. The obtained results depended on the species. The highest values were observed for *A. graveolens* and *P. crispum*. For *A. graveolens*, the best results were obtained for the F10 and F20 ferments in the lowest concentration. Similarly high results were observed for *P. crispum*, especially for the pure extract in the highest concentration of 5.0% and for F10 at 1.0%. For *D. carota*, most of the results were not significantly higher than the control, with the only exception of the F20 ferment at 0.5%. For all species, a high concentration combined with a long fermentation gave unwanted results. A cytotoxic effect was observed for F20 *D. carota*, F20 *A. graveolens* and F10 *P. crispum* (Figure 7).

**Figure 8:**
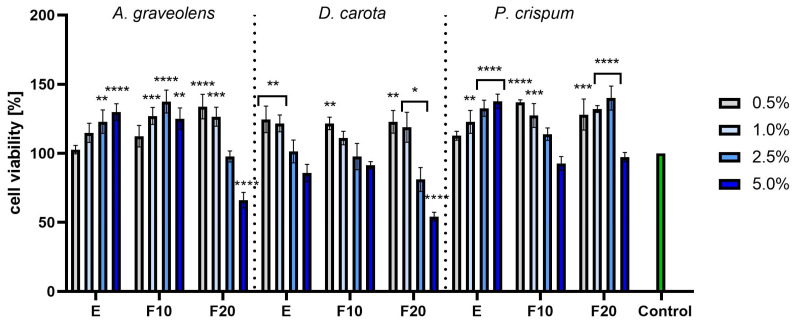
The effect of increasing concentrations *A. graveolens, D. carota* and *P. crispum* extracts and ferments on Neutral Red dye uptake in cultured fibroblasts after 24 h of exposure. Data are the means ± SD of three independent experiments in which each sample was tested in three replicates. **** *p* < 0.0001, *** *p* < 0.001, ** *p* < 0.01, * *p* < 0.05.

In the next step, cytotoxicity was assessed on the HDF cell line using the NR test, which showed a much stronger effect than the previously described test. For *A. graveolens*, the highest values were noted for extracts in the concentration of 5.0%, the F10 ferment in the concentration of 2.5% and the F20 ferment in the lowest concentration of 0.5%. Comparably high values were observed for *P. crispum*, with a similar dependency on the concentration. For the extracts, the best values were recorded for the highest investigated concentration of 5.0%, while for the F10 and F20 ferments the best values appeared for the lowest and moderate concentrations. *D. carota* showed a slightly worse effect on the viability of the tested cells compared to the two other plants tested. The carrot extract and ferments increased cell viability only when the samples were used at a concentration of 0.5% and 1.0%. Similar to previous experiments, here it has also been shown that a long fermentation along with a high concentration leads to a cytotoxic effect. This was especially strong for F20 *A. graveolens* and F20 *D. carota* (Figure 8).

**Figure 9:**
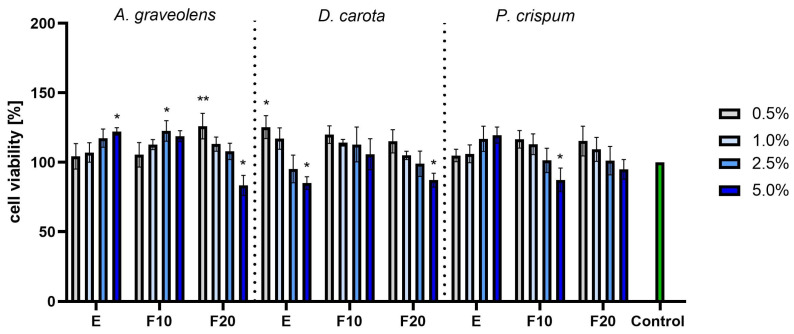
The effect of increasing concentrations of *A. graveolens*, *D. carota* and *P. crispum* extracts and ferments on Neutral Red dye uptake in cultured keratinocytes after 24 h of exposure. Data are the means ± SD of three independent experiments in which each sample was tested in three replicates. ** *p* = 0.0081, * *p* < 0.05.

In the last experiment, the lowest number of results remaining significantly higher than the control was observed. For *P. crispum*, no single case gave a significantly high result. For *D. carota*, the only significantly high value was observed for the extract in a concentration of 0.5%. For *A. graveolens*, statistically significant high values were observed for the extract in a concentration of 5.0%, the F10 ferment in the concentration of 2.5% and the F20 ferment in a concentration of 0.5%. The greatest cytotoxic effect was observed for the sample with a concentration of 5% and the longest fermentation time of F20. It was also observed that the cytotoxic effect increases with the combination of a high concentration of the ferments and a long fermentation time. The observed cytotoxic properties may also be driven by the low pH of the extracts and ferments in higher concentrations and the presence of compounds; therefore, during the studies, attempts were made to select the optimal concentrations for analysis (Figure 9).

Studies conducted by other researchers have also shown that the effect of *A. graveolens* and *D. carota* and *P. crispum* extracts significantly affects the viability of HaCaT and HDF cells and the integrity of the cell membrane, but with increasing doses the extracts can have cytotoxic effects [41,42]. Additionally, analyses have confirmed that the methanol extract of *P. crispum* has properties that inhibit cell proliferation and differentiation and also inhibits cell adhesion due to the presence of various polyphenols, such as gallic acid, acacetin and quinic acid [12,43,44].

In the studies conducted by Lantto, the effect of aqueous extract of *P. crispum* leaves on the survival of a human neuroblastoma cell line was examined. Analyses in the presence of *P. crispum* extract at concentrations of 0.01 ÷ 2 mg/mL did not induce a cytotoxic effect [45]. However, another recent study by Danciu et al. (2021) also showed an effect on tumor lines, showing a decrease in the viability of human breast adenocarcinoma cells (MCF7) after treatment with *P. crispum* extracts [46].

### 2.4. Assessment of Anti-Inflammatory Activity

**Figure 10:**
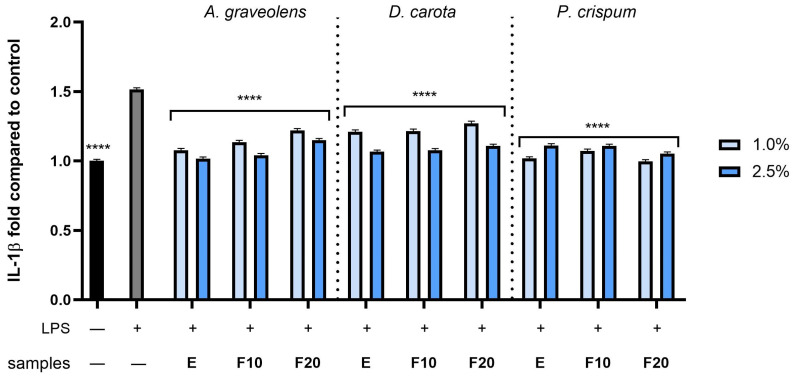
The effect of extracts and ferments of *A. graveolens*, *D. carota* and *P. crispum* roots after exposure to bacterial LPS (10 μg/mL) on the level of interleukin 1β calculated as a percentage in comparison with the untreated control. Data are mean ± SD from three independent experiments in which each sample was tested in duplicate. **** *p* < 0.0001.

**Figure 11:**
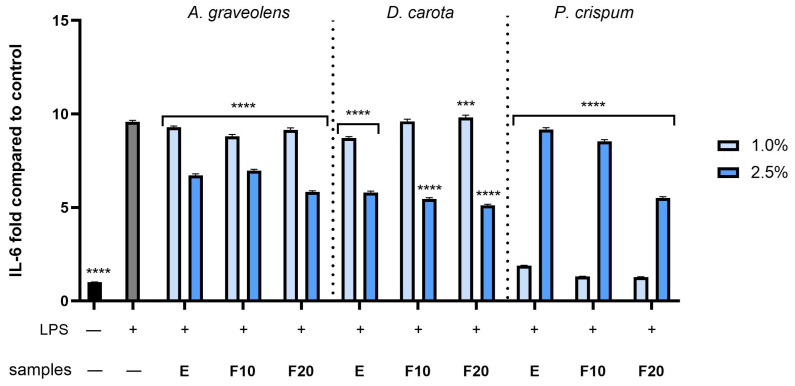
The effect of extracts and ferments of *A. graveolens*, *D. carota* and *P. crispum* roots after exposure to bacterial LPS (10 μg/mL) on the level of interleukin 6 calculated as a percentage in comparison with the untreated control. Data are mean ± SD from three independent experiments in which each sample was tested in duplicate. **** *p* < 0.0001, *** *p* = 0.0004.

**Figure 12:**
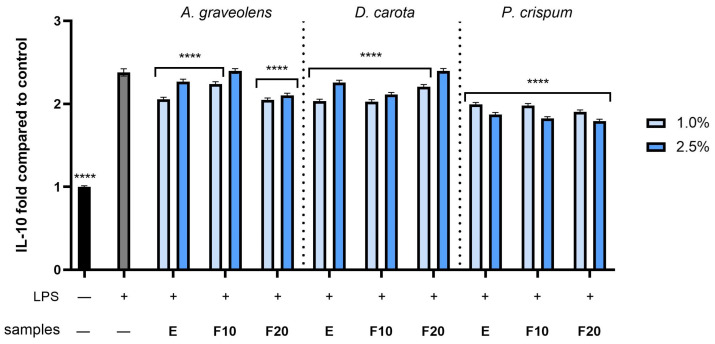
The effect of extracts and ferments of *A. graveolens*, *D. carota* and *P. crispum* roots after exposure to bacterial LPS (10 μg/mL) on the level of interleukin 10 calculated as a percentage in comparison with the untreated control. Data are mean ± SD from three independent experiments in which each sample was tested in duplicate. **** *p* < 0.0001.

To examinate the anti-inflammatory activity of *A. graveolens*, *D. carota* or *P. crispum* root extracts and kombucha ferments, the levels of pro-inflammatory interleukins (IL-1β and IL-6) as well as anti-inflammatory interleukins (IL-10) were monitored in human fibroblast cells treated with bacterial lipopolysaccharide (LPS). Results were expressed as the activity level of the cytokines tested relative to the control sample (cells untreated with compounds and LPS). As shown in Figure 10, Figure 11 and Figure 12, LPS is a potent inducer of cytokine production in HDF cells. The results showed that all compounds at both dilutions tested (1.0 and 2.5%) have the ability to reduce the pro-inflammatory IL-1β and IL-6 compared to the control (cells not treated with the compounds tested, but treated with LPS). In the case of the IL-1β level (Figure 10), the most favorable effect was observed for F10 (*A. graveolens*), F10 (*D. carota*) and F20 (*P. crispum*), obtaining 1.04 ± 0.012, 1.07 ± 0.013 and 0.99 ± 0.013 fold changes (compared to control without LPS and samples), respectively, at the dilutions tested: 2.5, 2.5 and 1.0%, respectively. According to the results shown in Figure 11, the level of interleukin 6 tested increased several times after LPS induction in the control sample as well as in some plant samples. As shown, both the extract and kombucha ferments from *P. crispum* at a dilution of 1.0% showed the highest ability to reduce interleukin 6, reaching 1.88 ± 0.022, 1.3 ± 0.015 and 1.27 ± 0.016 fold changes for E, F10 and F20, respectively. Moreover, F20 from *A. graveolens* and *D. carota* obtained the highest ability to reduce the level of IL-6 activity compared to E and F10 from the plants tested. The values were 5.88 ± 0.069 and 5.11 ± 0.061 fold compared to the control, respectively, at 2.5% dilution. This study also assessed the level of anti-inflammatory activity of interleukin 10, which is a key mediator in inhibiting the inflammatory response, in addition to reducing the production of pro-inflammatory cytokines such as IL-1β and IL-6. As shown in Figure 12, F10 and F20 at a 2.5% dilution from *A. graveolens* and *D. carota* obtained the most favorable effect on the activity of the cytokine tested, reaching 2.38 ± 0.028 and 2.37 ± 0.037 fold compared to the control, respectively. These results are strongly similar to the LPS-treated control sample.

It is worth mentioning that the presence of a number of active compounds (including phenolic acids and flavonoids; Table 1 and Table 2) in the tested extracts and ferments may contribute to the alleviation of inflammation. Flavonoids, known for their anti-inflammatory properties, can influence fibroblast activity by modulating cytokine production. Fibroblasts, the primary cells responsible for maintaining the integrity of connective tissue, play a key role in wound healing and tissue repair. However, their dysregulation can lead to excessive fibrosis and chronic inflammation. IL-1β and IL-6 are pro-inflammatory cytokines associated with various chronic inflammatory diseases. Flavonoids have been shown to inhibit the production of the above cytokines. IL-10, on the other hand, is an anti-inflammatory cytokine that helps regulate the immune responses. Some phenolic acids and flavonoids can increase IL-10 production, thereby promoting anti-inflammatory effects. For example, in a study on human dermal fibroblasts (NHDFs), kaempferol (100 nM) reduced the cytotoxicity and IL-1β expression induced by 12-O-tetradecanoylphorbol-13-acetate (TPA), which induces inflammation. The mechanism of action of kaempferol includes the blockade of reactive oxygen species (ROS) production, which prevents c-Jun N-terminal kinase (JNK) phosphorylation and inhibition of the NF-κB pathway. Inhibition of NF-κB and IκBα by kaempferol reduces activation of the inflammatory cascade, including caspase-3 expression and IL-1β secretion, confirming its role in reducing inflammation and cytotoxicity in dermal fibroblasts [47]. Furthermore, it was observed that apigenin, kaempferol, luteolin and quercetin, at concentrations of 50 and 100 nM, reduced IL-6 and TNF-α secretion levels in LPS-stimulated RAW 264.7 macrophages [48]. Furthermore, quercetin and luteolin exerted stimulatory effects on the expression of the anti-inflammatory cytokine IL-10, but at low concentrations (<50 μM) [49]. As is well known, carrot root is characterized by the presence of carotenoids. Crocin, a carotenoid compound, has been shown to significantly inhibit lipopolysaccharide (LPS)-induced expression of pro-inflammatory cytokines, including IL-6, in human fibroblast-like synoviocytes. One study showed that crocin achieves this by inhibiting the nuclear factor-kappa B (NF-κB) signaling pathway, which is crucial for the transcription of various inflammatory cytokines [49]. In addition, astaxanthin has demonstrated anti-inflammatory activity by reducing the expression of enzymes involved in the inflammatory process (iNOS, cyclooxygenase-COX, phospholipase A2 and hyaluronidase), inhibiting the transcription of nuclear factor (NF)-κB and reducing the expression of inflammatory cytokines (MCP1, TNF-α, IL-6 and IFN-γ). Similarly, violaxanthin inhibited the production of NO and prostaglandin E2 and the expression of NF-κB in LPS-stimulated macrophages [50].

Furthermore, many studies indicate the anti-inflammatory properties of the plants tested in this study. Methanolic celery root extract decreased AST, ALT, ALP, TNF-α and IL-1β levels and increased TAC and GSH in rats with acetaminophen-induced hepatotoxicity. Celery extract (300 mg/kg) was also shown to reduce IL-6 levels and caspase-3 expression in rats with lead-induced hepatitis. In another study, aqueous celery extract reduced levels of inflammatory cytokines (IL-6, IL-1β, IL-18 and TNF-α) in A-549 lung cells with LPS-induced damage, indicating its potential in alleviating inflammation [51,52,53]. Studies have shown that carrot polyacetylenes reduce IL-6 secretion in porcine aortic endothelial cells, reducing IL-6 protein levels by 77% at doses of 6.6 and 13.3 µg/mL. A dose-dependent reduction in NO production and mRNA and protein of pro-inflammatory cytokines (IL-6, IL-1β and TNF-α) was observed in macrophages [54]. Another study showed that carrot callus extract decreased COX-2 and TNF-α expression while increasing IL-10 in skin fibroblasts (HDFs) exposed to UV-B radiation [55]. In addition, 6-methoxymellin, a compound isolated from carrots, reduced the nuclear localization of nuclear factor-kappa B (NF-κB) subunits p65 and p50, leading to decreased mRNA transcription and secretion of IL-6 and IL-8 [56]. Moreover, studies have showed that myristicin, a compound found in parsley, can inhibit several cytokines and mediators responsible for the chemotaxis of the inflammatory process, such as tumor necrosis factor alpha (TNF-a), interleukins (IL-1, IL-6, IL-8, IL-10 and IL-17), nitric oxide (NO), macrophage inflammatory proteins (MIP-1α and MIP-1β), colony-stimulating factor (GM-CSF), IP-10, MCP-1 and MCP-3 and myeloperoxidase (MPO). This inhibition occurs both at the protein level and at the mRNA regulation level. In vitro studies have shown that the inhibition of these cytokines was able to block the migration and growth of neutrophils and macrophages, while in vivo it promoted a reduction in mice paw edema [57,58]. So far, there is a lack of research on kombucha tea fungus-fermented extracts of celery, carrot and parsley roots. Instead, research indicates that fermented celery (*A. graveolens*) shows significant anti-inflammatory properties. A study published by Dong et al. showed that probiotic fermentation of celery juice increases the content of its active compounds, including polyphenols, flavonoids, vitamin C and superoxide dismutase (SOD). In experiments with mice on a high-fat diet, consumption of fermented celery juice significantly inhibited weight gain, prevented dyslipidemia and hyperglycemia, inhibited visceral fat accumulation and favorably altered the composition of gut microflora [59].

### 2.5. Assessment of Antibacterial Activity

The next stage of this study included the assessment of the antibacterial activity of the tested extracts and ferments against several bacterial strains. The possibility of inhibiting the multiplication of these microorganisms by the tested samples is extremely important in the context of their potential use in products applied directly to the skin, as many skin lesions are accompanied by coexisting bacterial infections [60]. The conducted analyses assessed the effect of extracts and ferments on the growth of such bacteria as *Staphylococcus aureus*, *Staphylococcus epidermidis*, *Bacillus subtilis*, *Staphylococcus capitis*, *Micrococcus luteus*, *Yersinia enterocolitica* and *Pseudomonas aeruginosa*. All of the analyzed plants showed antibacterial activity dependent on the applied concentration which varied depending on the tested strains. The results of the conducted analyses indicated that the analyzed ferments were characterized by significantly stronger activity compared to the extracts. In most cases, the extension of fermentation time had a positive effect on the antibacterial properties of the tested samples, as the observed growth inhibition zones were larger and the minimum inhibitory concentration (MIC) values were lower (Table 3 and Table 4).

Considering the obtained results, among the three plants tested, the strongest antibacterial properties were observed for extracts and ferments from *D. carota*. In the case of this plant, only the extract did not inhibit the multiplication of *Y. enterocolitica* and *P. aeruginosa*, while the ferments were able to inhibit the growth of all seven bacterial strains tested. This effect was dependent on both the concentration of the extract or ferment used, as well as the fermentation time (Table 3). The MIC values for carrot ranged from 50–400 µg/mL (depending on the sample and bacteria used) and the growth inhibition zones exceeded 20 mm when *S. aureus*, *S. epidermidis* and *S. capitis* were exposed to the highest concentrations of F20 (Table 4). Extracts and ferments from parsley, on the other hand, showed the ability to inhibit the growth of six out of the seven strains tested, without causing inhibition of *Y. enterocolitica* growth. In the case of this plant, a stronger antibacterial effect was also observed for the obtained ferments compared to the extract. Only in the case of *B. subtilis* was no inhibition of the growth of this bacterium observed for F20 at any of the concentrations used. However, for most of the strains tested, the inhibition zone in the case of a 20-day ferment exceeded 20 mm (Table 3). The MIC values for parsley extract ranged from 150 to 700 µg/mL, while for ferments these values ranged from 100 to 200 µg/mL (Table 4). The weakest antimicrobial activity was demonstrated by samples obtained during the extraction and fermentation process of *A. graveolens* root. Celery extracts were able to inhibit four of the tested strains, reaching a maximum inhibition zone of 13 mm (in the case of using a 10% concentration on *S. capitis*). A slightly stronger effect was observed for ferments obtained from this plant, especially F20, which did not inhibit the growth of only B. subtilis. In the case of *S. aureus*, *S. capitis* and *M. luteus* strains, the growth inhibition zones after using the highest of the tested concentrations (10.0%) exceeded 20 mm (Table 3). The MIC values for celery extract ranged from 250 to 400 µg/mL, and for ferments they reached values from 200 to 400 µg/mL (Table 4).

The differences in the antibacterial activity of the tested samples are certainly related to the differences in the content of biologically active compounds. Taking into account the results of the chromatographic analysis, a correlation is visible between the content of the identified phytochemicals and the ability to inhibit the growth of various bacterial strains. The highest content of active compounds was noted in the carrot and parsley ferments, which explains the largest inhibition zones and the lowest MIC values obtained for these samples.

Antibacterial activity may be related to the content of compounds such as gallic acid. This compound can inhibit bacterial growth by changing the structure of the cell membrane, destroying cell walls and altering the metabolic activity of cells, as well as inhibiting the formation of bacterial biofilms [61,62,63]. Compounds such as gallocatechin, epigallocatechin, epicatechin and catechin may also play an important antibacterial role. As shown by the available literature data, these compounds can disrupt bacterial protein synthesis, change bacterial cell morphology and inhibit the formation of extracellular polysaccharide involved in biofilm formation [64,65]. Chlorogenic acid may also play an important role in the antibacterial activity of the obtained extracts and ferments, which may affect the increase in the permeability of the outer and plasma membrane of bacteria, resulting in the loss of the barrier function and a slight leakage of nucleotides. It has also been shown that this compound can disrupt multiple physiological pathways of bacterial cells, including those related to biofilm formation and increased membrane permeability. These changes may lead to bacterial cell death [66,67]. Quercetin and its derivatives are also antibacterial compounds. These phytochemicals can inhibit the growth of microorganisms by changing the permeability of bacterial cells, destroying their cell walls, inhibiting the synthesis of nucleic acids and enzyme activity and also influencing the expression of various proteins [68,69].

Analyses conducted in this work indicate for the first time the promising antibacterial properties of ferments obtained from root vegetables obtained by fermentation with kombucha. In the available literature, there are few reports on the fermentation of *A. graveolens*, *D. carota* and *P. crispum* roots to obtain products with antibacterial activity. The literature data only indicate the antibacterial activity of ferments from carrot by-products, which after fermentation with seven strains belonging to the genus *Lactobacillus*, *L. plantarum*, *L. casei*, *L. paracasei* and *L. rhamnosus* show multidirectional antimicrobial activity against bacteria such as *L. monocytogenes*, *E. coli*, *Salmonella* spp., *S. aureus* and *B. cereus* [70]. Other authors indicate a strong antibacterial effect of carrot HCl-ethanol peel extract, which has the ability to inhibit the growth of both Gram-positive bacteria (such as *Staphylococcus aureus* and *Bacillus cereus*) and Gram-negative bacteria (*Salmonella typhi* and *Escherichia coli*) at a minimum inhibitory concentration of 20 μg/mL [71]. Dedieu et al. also indicated the antibacterial potential of *D. carota* essential oil against Campylobacter [72]. There are no reports on the antibacterial activity of parsley ferments. Linde et al. indicated, however, that the essential oil obtained from this plant exhibits both bacteriostatic and bactericidal effects on bacteria such as *S. aureus*, *L. monocytogenes*, *S. enterica*, *B. cereus*, *E. cloacae*, *E. coli* and *P. aeruginosa* [73]. Other authors also indicated the antibacterial activity of the essential oil extracted from the aerial part of *P. crispum*. Their analyses indicated that this oil can inhibit the growth of bacteria such as *S. aureus*, *E. coli*, *B. cereus*, *S. typhi* and *S. epidermidis* [74]. Nouioura et al. also indicated the possibility of inhibition of the multiplication of both G-negative bacteria (such as *E. coli* and *S. enterica*) and G-positive bacteria (such as *S. aureus* and *B. subtilis*) by volatile compounds from parsley. These authors also indicated the possibility of the multiplication of the fungal strain Candida albicans [75]. There are also no literature reports on the antibacterial activity of *A. graveolens* ferments. There are only articles describing the antibacterial properties of extracts from this plant, mainly its aerial parts. Emad et al. indicated the possibility of inhibiting the multiplication of *E. coli* and *S. aureus* by both extracts from celery aerial parts and by the dichloromethane fraction obtained from this plant [76]. Sunarno et al. also indicate the strong potential of the ethanol extract obtained from powdered celery leaves and stems against *S. aureus*, indicating the possibility of its incorporation into the structures of hydrogels based on hydroxypropylmethylcellulose [77]. Alves-Silva et al. showed that essential oils extracted from the whole plant (stems, leaves and flowers) had the ability to inhibit the growth of bacterial strains such as *A. hydrophila*, *P. fragi*, *A. denitrificans*, *S. marcenscens*, *S. putrefaciens*, *A. faecalis*, *E. amnigenus* and *E. gergoviae*. They also demonstrated the ability of these oils to inhibit the activity of various yeast strains such as *S. cerevisiae*, *C. zeylanoides*, *Y. lipolytica*, *D. hansenii* and *P. caronii* [78]. In summary, our work indicates for the first time the high antibacterial potential of bioferments obtained from the roots of *A. graveolens*, *D. carota* and *P. crissum* by fermentation using kombucha. The significantly higher antibacterial activity of the obtained ferments in comparison with the analyzed extracts indicates the validity of fermenting plant extracts in order to increase their biological activity.

Due to the fact that the fermentation products of the plants studied in this work are a rich source of many antibacterial compounds, they can be seen as natural preservatives with potential use in cosmetic and pharmaceutical products. Their inclusion in the formulation of various cosmetic formulations could reduce the concentration of synthetic preservatives in these products. However, it should be remembered that in order to ensure the safety of these natural preservatives, these ferments should be purified from bacterial cells and should be assessed in terms of the safety of their use, the presence of individual phytochemicals and the effectiveness of the developed preservative system.

## 3. Materials and Methods

### 3.1. Plant Material, Extraction and Fermentation Procedure

For the analyses, the roots of *A. graveolens*, *D. carota* and *P. crispum* were obtained from a local producer. The starter culture of the kombucha tea fungus (SCOBY) was purchased from a commercial supplier (Nasza Przyszłość, Tapin, Poland). To prepare the extracts, 3 g of celery, carrot and parsley roots were added to 100 mL of distilled water in separate beakers. The extraction process was carried out using a magnetic stirrer for 24 h, after which the extracts were sonicated in an ultrasonic bath (Digital Ultrasonic Cleaner, Thermo Fisher Scientific, Waltham, MA, USA) for 30 min at room temperature. The temperature during extraction varied in the range of 23.5–26.1 °C. The obtained extracts were then subjected to fermentation with kombucha. For this purpose, 200 mL samples of all three extracts were poured into separate 1000 mL sterile beakers. The solutions were supplemented with sucrose and kombucha at a final concentration of 10.0% *w/v*. The fermentation was carried out for 10 and 20 days, respectively, in separate beakers at room temperature (approximately 25 °C), avoiding direct exposure to sunlight. The ferments collected after 10 and 20 days were labeled as F10 and F20, respectively, and the unfermented extract was labeled as E. For the analyses, the obtained extracts and ferments were diluted 200, 100, 40 and 20× and labelled as 0.5%, 1.0%, 2.5% and 5.0%, respectively.

### 3.2. Determination of Biologically Active Compounds

All standards, as well as MS-grade reagents such as formic acid and acetonitrile, were obtained from Sigma-Aldrich (St. Louis, MO, USA).

The metabolites were identified using an ultra-high-performance liquid chromatography (UHPLC) system from the Infinity Series II, equipped with a DAD detector and an Agilent 6224 ESI/TOF mass detector (Agilent Technologies, Santa Clara, CA, USA). The chromatographic setup included a Kinetex C18 reversed-phase column (Phenomenex, Torrance, CA, USA) with dimensions of 150 mm in length, 2.1 mm inner diameter and a particle size of 1.7 µm. The column thermostat was maintained at 30 °C and the flow rate was set to 0.2 mL/min. The mobile phase consisted of water with 0.05% formic acid (solvent A) and acetonitrile with 0.05% formic acid (solvent B). Compound separation was achieved via gradient elution with the following program: 0–8 min with 98% A transitioning to 93% A, 8–15 min with 93% A transitioning to 88% A, 15–29 min with 88% A transitioning to 85% A, 29–40 min with 85% A transitioning to 80% A and 40–80 min with 80% A transitioning to 55% A. Chromatograms were obtained across a wavelength range of 200–600 nm. For LC–MS analysis, the ion source was configured with the following parameters: a drying gas temperature of 325 °C, a flow rate of 8 L/min, a nebulizer pressure of 30 psi, a capillary voltage of 3500 V, a fragmentor voltage of 220 V and a skimmer voltage of 65 V. Ion detection was performed within a mass spectrometer detector. MS identification was conducted by comparing the results with available standards or referencing literature data in cases where standards were unavailable. Quantification was carried out using calibration curves prepared from standard solutions of the identified compounds.

### 3.3. Determination of Antioxidant Properties

#### 3.3.1. ABTS Scavenging Assay

The antioxidant properties of the tested extracts and ferments from roots of *A. graveolens, D. carota* and *P. crispum* were assessed using the ABTS Scavenging Assay described previously by Miller et al. [79]. The first step was to prepare a mixture of 7 mM ABTS solution (Merck, Darmstadt, Germany) and 2.4 mM potassium persulfate (Warchem, Zakret, Poland) in a 1:1 ratio. The mixture prepared in this way was left at room temperature of about 22 °C for 14 h. After this time, the solution was diluted in PBS until the absorbance value of 1.0 ± 0.04 at λ = 734 nm was obtained. In the next step, the tested samples (in the concentration range of 0.5%, 1.0%, 2.5% and 5.0%) were mixed thoroughly with the previously prepared ABTS solution, and then absorbance measurements of the tested samples were taken at λ = 734 using a UV/VIS spectrophotometer (Thermo Fisher Scientific, Waltham, MA, USA). Trolox (Merck KGaA, Darmstadt, Germany) and ascorbic acid (Warchem, Zakret, Poland) were used as positive controls with proven antioxidant activity, distilled water as a negative control and individual extracts/ferments without ABTS as blanks. Results are presented as percentage of ABTS scavenging compared to the control (Equation (1)). Three independent experiments were performed, each with triplicate measurements at each concentration.

(1) $$\%\;ABTS\;scavenging=\left(1-\left(\frac{Abs\;sample}{Abs\;control}\right)\right)\times 100$$

#### 3.3.2. DPPH (1,1-Diphenyl-2-picrylhydrazyl) Radical Scavenging Assay

The antioxidant properties of the celery root, carrot and parsley extracts and ferments were assessed using the DPPH (1,1-diphenyl-2-picrylhydrazyl) radical assay, based on the method previously described by Brand-Williams et al. [80] with some modifications. For this purpose, samples of the extracts and ferments at concentrations of 0.5%, 1.0%, 2.5% and 5.0% were applied to 96-well plates. Then, 100 µL of 4 mM DPPH methanol solution (Merck KGaA, Darmstadt, Germany) was added to each well and mixed thoroughly on an orbital shaker. Trolox and ascorbic acid were used as positive controls, distilled water as a negative control and individual extracts/ferments without DPPH as blanks. The plate with the samples was placed in a plate reader and absorbance was measured at λ = 517 nm every 5 min for 20 min. The results are presented as a percentage of DPPH scavenging compared to the control (Equation (2)). Three independent experiments were performed, each with triplicate measurements at each concentration.

(2) $$\%\;DPPH\;scavenging=\frac{Abs\;control-Abs\;sample}{Abs\;control}\times 100$$

#### 3.3.3. Detection of Intracellular Levels of Reactive Oxygen Species (ROS)

To assess the potential of *A. graveolens, D. carota* and *P. crispum* roots extracts and ferments to reducing intracellular reactive oxygen species (ROS) production in skin cells, the method described by Grauzdytė et al. was used [81]. The fluorogenic probe H_2_DCFDA was used for this purpose. Human skin cells, HDFs and HaCaTs, were seeded at a density of 1 × 10^4^ on 96-well plates and incubated for 24 h. Following incubation, DMEM was replaced with the tested extracts and ferments dissolved in DMEM (in the concentration range of 0.5%, 1.0%, 2.5% and 5.0%) and incubated again in the incubator for 24 h. After this time, DMEM (Dulbecco’s Modified Eagle Medium) was removed and cells were treated with 10 μM H_2_DCFDA (Sigma Aldrich, Sant Louis, MO, USA) dissolved in DMEM without FBS serum. Hydrogen peroxide (H_2_O_2_) was then added to each well to achieve a final concentration of 500 μM. Cells exposed to 500 μM hydrogen peroxide (H_2_O_2_, Merck KGaA, Darmstadt, Germany) served as positive controls, while cells treated without test extracts or H_2_O_2_ served as negative controls, both for HDF and HaCaT cells. After 60 min of incubation, fluorescence measurements were performed at an excitation wavelength of λ = 485 nm and an emission wavelength of λ = 530 nm using a microplate reader (FilterMax F5, Thermo Fisher Scientific, Waltham, MA, USA). Three independent experiments were performed for the analyses and each sample was tested in triplicate.

### 3.4. Cytotoxicity Analysis

#### 3.4.1. Cell Culture

Two cell lines were used to evaluate cytotoxicity and intracellular reactive oxygen species levels: fibroblasts (HDFs) and keratinocytes (HaCaTs), both obtained from CLS Cell Lines Service (Eppelheim, Germany). Both cell types were cultured in high-glucose DMEM (VWR International, Radnor, PA, USA) supplemented with 1% antibiotics, including100 U/mL penicillin and 1000 μg/mL streptomycin (Thermo Fisher Scientific, Waltham, MA, USA) and 10% fetal bovine serum (FBS, from VWR International, Radnor, PA, USA). Cells were cultured in culture flasks under controlled conditions of 37 °C with 5% CO₂ and 95% air in a humidified incubator. After the cells reached 70–80% confluence, the culture medium was removed and the cells were washed twice with PBS (Phosphate Buffered Saline, Biological Industries, Kibbutz Beit-Haemek, Israel). Afterwards, the cells were then trypsinized and placed in fresh DMEM. The cells were seeded in 96-well plates at a density of 1 × 10^4^ cells/well and incubated for 24 h to prepare the cells for cytotoxicity assays.

#### 3.4.2. Alamar Blue

The Alamar Blue Assay was the first method used to evaluate skin cell viability, following the protocol described by Page et al. [82] with modifications. After a 24 h incubation of skin cells in 96-well plates, the medium was replaced with the tested extracts and ferments (at concentrations of 0.5%, 1.0%, 2.5% and 5.0%) and incubated for another 24 h. Subsequently, a 60 µM resazurin solution (Merck KGaA, Darmstadt, Niemcy) was added to each well. Untreated cells served as controls. The plates were incubated for 2 h, after which fluorescence was measured at λ = 570 nm using a microplate reader (Thermo Fisher Scientific, Waltham, MA, USA). Three independent experiments were performed, each with triplicate measurements for each concentration.

#### 3.4.3. Neutral Red

The Neutral Red Assay was the second method used to evaluate skin cell viability, following the procedure described by Borenfreund et al. [83]. After 24 h of incubation in 96-well plates, the culture medium was replaced with the tested extracts and ferments at concentrations of 0.5%, 1.0%, 2.5% and 5.0%, followed by another 24 h incubation. After 24 h of incubation of the skin cells with the tested extracts and ferments from celery, carrot and parsley roots, Neutral Red dye (Merck KGaA, Darmstadt, Germany) dissolved in DMEM was introduced into each well. HDFs and HaCaTs were incubated with the dye for 2 h. Subsequently, the Neutral Red dye was aspirated, the cells were washed with sterile PBS and a decolorizing solution (containing ethanol/acetic acid/water in proportions of 50%/1%/49%) was added. Untreated cells served as controls. Absorbance were taken using a microplate reader at λ = 540 nm. Three independent experiments were performed, each with triplicate measurements for each concentration.

### 3.5. Assessment of Anti-Inflammatory Activity

To evaluate the anti-inflammatory effects of extracts and ferments from roots of *A. graveolens*, *D. carota* and *P. crispum*, the levels of IL-1β, IL-6 and IL-10 were measured in fibroblasts (HDFs) exposed to bacterial lipopolysaccharide (LPS, 10 µg/mL) derived from Escherichia coli O111:B4 for 24 h. Simultaneously, the cells in 6-well plates were treated with all tested extracts and ferments at concentrations of 1.0% and 2.5%. After a 24 h incubation of cells treated with LPS and the tested extracts and ferments from celery, carrot and parsley roots, DMEM was removed from the wells. The wells were washed with PSB and then 150 µL of RIPA buffer was added to each well, which caused cell lysis. The cells prepared in this way were analyzed using commercially available ELISA kits (Elabscience Biotechnology Inc., Houston, TX, USA), according to the manufacturer’s instructions. Absorbance was measured at a wavelength of 450 nm using a microplate reader (FilterMax F5, Thermo Fisher Scientific, Waltham, MA, USA). The cells not treated with LPS or extracts and ferments were used as a negative control, while cells treated with LPS but not treated with extracts and ferments were used as a positive control.

### 3.6. Assessment of Antibacterial Activity

#### 3.6.1. Disk Diffusion Assay

The disk diffusion method was used to assess the effect of the analyzed extracts and ferments on the growth of pathogenic bacteria. The analyzed bacterial strains were obtained from the American Type Culture Collection (Manassas, VA, USA). The antibacterial properties of the tested samples were assessed using the following strains: *Staphylococcus aureus* ATCC BAA-2312, *Staphylococcus epidermidis* ATCC^®^ 49134™, *Bacillus subtilis* ATCC^®^ 19659, *Staphylococcus capitis* ATCC^®^ 146™, *Micrococcus luteus* ATCC^®^ 10240™, *Yersinia enterocolitica* ATCC 27729 and *Pseudomonas aeruginosa* ATCC^®^ 35032. First, 10 mL of agar medium, appropriate for each bacterial strain, was poured onto sterile Petri dishes. The media used in the study were tryptone soy agar, nutrient agar, LB agar, cetrimide agar and MRS agar. All culture media were purchased from Argenta (Poznań, Poland). Then, individual bacterial strains were inoculated onto separate Petri dishes at a density of 5 × 10^7^ CFUs (colony forming units)/mL. Dilutions of the tested samples were also prepared (1.0, 5.0 and 10.0%) and the solutions prepared in this way were sterilized through membrane filters (0.22 µm). In the next step, sterile filter paper discs were soaked with the tested compounds and then placed on the surface of a solidified agar medium on which bacteria had been previously inoculated. A sterile disc soaked in sterile distilled water was used as a negative control, while appropriate antibiotics inhibiting the growth of the tested strains were used as a positive control. The dishes prepared in this way were placed for 2 h in a refrigerator at 4 °C to allow the tested extracts and ferments to initially diffuse in the medium. Then, the prepared plates were transferred to an incubator and cultured at 35 ± 2 °C for 24 h. After this time, the ability of growth inhibition was assessed by measuring the diameter of the inhibition zone.

#### 3.6.2. Determination of Minimum Inhibitory Concentrations (MIC)

The antibacterial activity of extracts and ferments from the roots of *A. graveolens*, *D. carota* and *P. crispum* against the seven analyzed bacterial strains was also assessed by determining the minimum inhibitory concentration (MIC). For this test, samples of extracts and ferments were evaporated and dissolved in sterile distilled water to obtain stocks at a concentration of 10 mg/mL. To determine the MIC, the microdilution technique in broth with p-iodonitrotetrazolium violet (INT, Merck KGaA, Darmstadt, Germany) as a growth indicator was used, which was previously described by Eloff [84]. First, the analyzed extracts and ferments were diluted multiple times with broth to obtain concentrations from 25 to 2000 µg/mL. Then, the prepared sample dilutions (in triplicate) were placed in 96-well microplates. In the next step, a suspension of the tested bacterial strain (containing 5 × 10^4^ CFUs) was added to each well. For each of the tested bacterial strains, a sterility control well, an appropriate antibiotic well and a growth control well were also prepared. The plates were then incubated in an incubator at 37 °C for 24 h. After this time, 40 μL of INT solution (0.4 mg/mL) was added to each well of a 96-well plate. The plates were then placed in an incubator at 37 °C for 30 min. The MIC value was determined as the concentrations of the tested extracts and ferments that inhibited the total growth of individual bacterial strains. The study consisted of three independent experiments, in which each sample was tested in triplicate.

### 3.7. Statistical Analysis

The values of the analyzed parameters are presented as mean ± standard deviation (SD). At the beginning, it was checked whether the data obtained during the analyses met the assumption of normality and were subject to a normal distribution by performing the Shapiro–Wilk test. Statistical significance was assessed using two-way analysis of variance (ANOVA) followed by Dunnett’s post hoc test for group comparisons. The variance was related to the interaction between the experimental factors such as the type of sample used and its concentration. Statistical significance was considered at **** *p* < 0.0001, *** *p* < 0.001, ** *p* < 0.01 and * *p* < 0.05 compared to the control group. Statistical analyses were performed using GraphPad Prism 8.4.3 (GraphPad Software, Inc., San Diego, CA, USA).

## 4. Conclusions

The conducted studies have shown that the analyzed ferments are characterized by an increased level of biologically active compounds compared to the aqueous extracts. The ferments were characterized by antioxidant, anti-inflammatory and antibacterial properties, better than the analyzed extracts. The ferments had better free radical scavenging capacity and reduced ROS levels in cells compared to the extracts, which was probably due to the presence of more phytochemicals that were detected during chromatographic analyses. The stronger antibacterial activity observed for the obtained bioferments indicates that plant extracts fermented with kombucha can be a valuable source of substances with preservative activity in various types of cosmetic preparations. Additionally, it was shown that the tested extracts and ferments at concentrations up to 2.5% (except F20) do not cause cytotoxicity to skin cells (keratinocytes and fibroblasts). Furthermore, it was observed that all the plant samples tested have the ability to reduce the levels of the cytokines tested. The conducted research confirmed that the analyzed *Apiaceae* bioferments contain a number of bioactive substances that have a positive effect on skin and may be a valuable active ingredient in cosmetic products. In particular, the bioferment obtained from *D. carota* roots deserves attention, as it is a raw material approved for use in cosmetic products and is fermented with lactic acid bacteria of the *Lactobacillus* and *Bifidobacterium* spp. It was therefore reasonable to carry out fermentation using kombucha. Studies have confirmed its antioxidant, anti-inflammatory and antimicrobial properties. However, it should be emphasized that, despite the promising results indicating significant biological activity, an extended toxicological analysis is needed to comprehensively assess the safety of the potential cosmetic raw material. In addition, the fermentation process is dependent on a number of factors, such as the concentration of the plant raw material and sugars, the temperature and the time of fermentation. Consequently, standardization of this method can be a significant challenge, especially in terms of ensuring reproducibility of the microbial composition, including the stability of bacterial and fungal populations. Despite these challenges, the results obtained show the promising prospect of using fermentation processes to obtain bioferments with potential applications in the cosmetics industry.

## Figures and Tables

**Table 1:** Mass data of polyphenols found in fermented extract from the roots of *P. crispum*.

R_T_ (min.)	Mass Data (*m/z*-H)	Fragment (*m/z*-H)	Formula	Δppm	Component
4.88	169.01502	(125)	C_7_H_6_O_5_	4.55	Gallic acid
6.24	343.06757	(169, 191)	C_14_H_16_O_10_	1.45	Galloylquinic acid
7.18	343.06788	(169, 191)	C_14_H_16_O_10_	2.35	Galloylquinic acid
9.60	305.06768		C_15_H_14_O_7_	3.28	Gallocatechin
11.53	353.08886	(179, 191)	C_16_H_18_O_9_	2.98	Neochlorogenic acid
14.61	305.06777		C_15_H_14_O_7_	3.57	Epigallocatechin
15.84	289.07122	(245, 221)	C_15_H_14_O_6_	−1.87	Catechin
15.98	593.13076	(289)	C_30_H_26_O_13_	1.17	Procyanidin
16.79	353.08859	(135, 179, 191)	C_16_H_18_O_9_	2.21	Chlorogenic acid
19.72	289.07157	(245, 221)	C_15_H_14_O_6_	−0.66	Epicatechin
21.44	457.07744	(125, 169)	C_22_H_18_O_11_	−0.43	Gallocatechin gallate
23.97	457.07603	(125, 169)	C_22_H_18_O_11_	−3.50	Epigallocatechin Gallate
25.11	563.14333		C_26_H_28_O_14_	4.79	Unknown flavonoid
25.87	625.14049	(316, 479)	C_27_H_30_O_17_	−0.85	Unknown flavonoid
26.61	479.08291	(316)	C_21_H_20_O_13_	−0.43	Unknown flavonoid
28.15	771.20112	(300)	C_33_H_40_O_21_	2.83	Quercetin derivative
28.59	593.15317	(285)	C_27_H_30_O_15_	3.33	Kaempferol derivative
29.87	577.15794	(269, 431)	C_27_H_30_O_14_	2.87	Apigenin derivative
30.29	771.19823	(300)	C_33_H_40_O_21_	−0.91	Quercetin derivative
31.04	441.08345	(125, 169, 289)	C_22_H_18_O_10_	1.65	epicatechin gallate
31.39	577.15903	(269)	C_27_H_30_O_14_	4.76	Apigenin derivative
32.12	755.2036	(285)	C_33_H_40_O_20_	−0.55	Kaempferol derivative
32.37	609.1469	(300)	C_27_H_30_O_16_	1.3	Rutoside (str)
32.74	463.08716	(300)	C_21_H_20_O_12_	−2.24	Quercetin 3-galactoside (str)
33.34	441.08105	(169, 289)	C_22_H_18_O_10_	−3.78	Epicatechin gallate
33.87	463.0887	(300)	C_21_H_20_O_12_	1.08	Quercetin 3-glucoside (str)
35.87	755.20741	(285)	C_33_H_40_O_20_	4.49	Kaempferol derivative
37.40	447.09303	(284)	C_21_H_20_O_11_	−0.57	Kaempferol hexoside
38.28	593.15134	(284)	C_27_H_30_O_15_	0.25	Kaempferol 3-rutinoside (str)
39.55	447.09222	(284)	C_21_H_20_O_11_	−2.38	Kaempferol 3-glucoside (astragalin) (str)
40.03	563.14345	(269)	C_26_H_28_O_14_	5.0	Apigenin-7-apioglucoside (Apiin)
40.16	431.09698	(269)	C_21_H_20_O_10_	−3.22	Apigenin 7-glucoside (str)
51.55	301.20232		C_16_H_30_O_5_	0.9	Hydroxyhexadecanedioic acid
51.99	301.03545		C_15_H_10_O_7_	0.24	Quercetin (str)
58.92	269.04601		C_15_H_10_O_5_	1.17	Apigenin (str)
59.0	327.21922		C_18_H_32_O_5_	4.64	Trihydroxyoctadecadienoic acid
61.93	329.23488		C_18_H_34_O_5_	4.64	Trihydroxyoctadecenoic acid (Pinellic acid)
75.17	311.22377		C_18_H_32_O_4_	3.16	Octadecenedioic acid

str—identification was confirmed based on standard.

**Table 2:** Results of the quantitative analysis (±SD) of polyphenols in fermented extracts from *A. graveolens*, *D. carota* and *P. crispum* roots.

Component	*A. graveolens* (µg/mL ± SD)		*D. carota* (µg/mL ± SD)		*P. crispum* (µg/mL ± SD)	
	F10	F20	F10	F20	F10	F20
Gallic acid (str)	2.81 ± 0.30	2.92 ± 0.28	2.48 ± 0.02	3.27 ± 0.11	3.09 ± 0.05	7.31 ± 0.39
Galloylquinic acids ^1^	3.37 ± 0.08	3.41 ± 0.15	2.89 ± 0.03	3.25 ± 0.18	3.25 ± 0.10	3.40 ± 0.14
Gallocatechin ^2^	0.82 ± 0.04	0.78 ± 0.04	0.52 ± 0.03	0.51 ± 0.02	0.60 ± 0.03	0.63 ± 0.02
Epigallocatechin ^2^	0.88 ± 0.03	0.89 ± 0.04	1.81 ± 0.09	1.97 ± 0.09	1.90 ± 0.10	2.40 ± 0.11
Catechin (str)	1.07 ± 0.09	0.80 ± 0.06	1.76 ± 0.11	1.36 ± 0.10	1.77 ± 00.11	1.38 ± 0.10
Chlorogenic acids (str)	1.01 ± 0.01	0.97 ± 0.03	0.94 ± 0.05	1.02 ± 0.01	1.10 ± 0.40	1.10 ± 0.01
Epicatechin (str)	3.76 ± 020	4.12 ± 0.14	8.51 ± 0.31	8.76 ± 0.01	7.68 ± 0.36	8.74 ± 0.10
Epigallocatechin gallates ^1^	1.50 ± 0.13	0.30 ± 0.02	8.22 ± 0.25	8.18 ± 0.20	8.02 ± 0.37	4.15 ± 0.17
Flavonoid *m*/*z*-H= 563 ^3^	0.73 ± 0.01	0.75 ± 0.01	0.69 ± 0.01	0.78 ± 0.02	0.75 ± 0.01	0.85 ± 0.05
Flavonoid *m*/*z*-H = 625 ^3^	0.26 ± 0.01	0.27 ± 0.01	0.59 ± 0.04	0.62 ± 0.03	0.41 ± 0.02	0.46 ± 0.03
Flavonoid *m*/*z*-H = 479 ^3^	0.23 ± 0.01	0.25 ± 0.01	0.64 ± 0.03	0.74 ± 0.03	0.70 ± 0.02	0.74 ± 0.03
Quercetin derivatives *m*/*z*-H =771 ^3^	3.34 ± 0.20	3.51 ± 0.26	3.24 ± 0.19	3.74 ± 0.17	3.50 ± 0.12	3.81 ± 0.11
Kaempferol derivative *m*/*z*-H = 593 ^4^	1.11 ± 0.05	1.12 ± 0.05	1.04 ± 0.04	1.22 ± 0.04	1.21 ± 0.08	1.21 ± 0.07
(Epi)catechin gallates^1^	0.40 ± 0.02	0.01 ± 0.01	1.10 ± 0.05	1.18 ± 0.06	1.27 ± 0.06	0.33 ± 0.02
Kaempferol derivatives *m*/*z*-H = 755 ^4^	1.48 ± 0.06	1.51 ± 0.11	1.32 ± 0.09	1.50 ± 0.08	1.48 ± 0.08	1.53 ± 0.10
Rutoside (str)	1.01 ± 0.04	1.07 ± 0.06	0.98 ± 0.04	1.09 ± 0.05	1.04 ± 0.05	1.09 ± 0.05
Quercetin galactoside (str)	0.35 ± 0.02	0.36 ± 0.02	0.34 ± 0.01	0.40 ± 0.02	0.35 ± 0.02	0.34 ± 0.01
Quercetin glucoside (str)	0.41 ± 0.02	0.39 ± 0.03	0.40 ± 0.02	0.44 ± 0.03	0.46 ± 0.03	0.43 ± 0.03
Kaempferol rutinoside (str)	0.45 ± 0.03	0.47 ± 0.02	0.38 ± 0.02	0.44 ± 0.03	0.26 ± 0.01	0.39 ± 0.02
Kaempferol glucoside (str)	0.21 ± 0.01	0.22 ± 0.02	0.20 ± 0.02	0.23 ± 0.02	0.21 ± 0.01	0.24 ± 0.02
Apigenin-7-apioglucoside ^5^	0.28 ± 0.05	0.27 ± 0.02	n.d.	n.d.	3.17 ± 0.31	3.73 ± 0.25
Apigenin 7-glucoside (str)	0.07 ± 0.02	0.06 ± 0.02	n.d.	n.d.	0.17 ± 0.01	0.29 ± 0.02
Quercetin (str)	0.16 ± 0.02	0.16 ± 0.01	0.16 ± 0.01	0.17 ± 0.01	0.18 ± 0.01	0.17 ± 0.01
Apigenin (str)	det.	det.	det.	det.	det.	det.

^1^—quantification was based on calibration curve for gallic acid; ^2^—quantification was based on calibration curve for catechin; ^3^—quantification was based on calibration curve for rutoside, ^4^—quantification was based on calibration curve for kaempferol rutinoside, ^5^—quantification was based on calibration curve for apigenin 7-glucoside and str—quantification was based on calibration curve for standard.

**Table 3:** Antibacterial activity of the tested *A. graveolens*, *D. carota* and *P. crispum* root extract and ferments (F10 and F20), expressed as the diameter of the average inhibition zone (mm).

Bacteria	Plant Species	Zone of Inhibition [mm]								
		E			F10			F20		
		1.0%	5.0%	10.0%	1.0%	5.0%	10.0%	1.0%	5.0%	10.0%
*Staphylococcus aureus*	*A. graveolens*	4	6	7	5	6	8	10	17	20
	*D. carota*	nd	4	6	6	8	14	10	14	21
	*P. crispum*	nd	nd	4	5	7	10	10	15	19
*Staphylococcus epidermidis*	*A. graveolens*	3	5	7	10	13	14	9	15	17
	*D. carota*	7	8	12	10	15	20	14	19	24
	*P. crispum*	5	9	12	10	12	14	9	13	21
*Bacillus subtilis*	*A. graveolens*	5	7	10	nd	nd	nd	nd	nd	nd
	*D. carota*	6	11	17	4	8	12	5	8	13
	*P. crispum*	6	8	12	9	13	17	nd	nd	nd
*Staphylococcus capitis*	*A. graveolens*	7	11	13	11	15	18	12	18	24
	*D. carota*	5	8	11	8	14	16	13	18	21
	*P. crispum*	11	16	21	11	18	23	13	18	26
*Micrococcus luteus*	*A. graveolens*	nd	nd	nd	8	11	15	9	16	22
	*D. carota*	4	7	10	6	10	13	6	9	11
	*P. crispum*	6	8	13	7	11	17	9	15	22
*Yersinia enterocolitica*	*A. graveolens*	nd	nd	nd	nd	nd	nd	5	9	12
	*D. carota*	nd	nd	nd	4	7	9	6	10	12
	*P. crispum*	nd	nd	nd	nd	nd	nd	nd	nd	nd
*Pseudomonas aeruginosa*	*A. graveolens*	nd	nd	nd	6	10	14	8	12	15
	*D. carota*	nd	nd	nd	5	7	12	8	13	16
	*P. crispum*	4	7	9	5	10	13	10	16	21

nd—not detected.

**Table 4:** Minimum inhibitory concentrations (MIC) of *A. graveolens*, *D. carota* and *P. crispum* roots extract and ferments (F10 and F20) against the tested bacteria.

Bacteria	Plant Species	Minimum Inhibitory Concentration MIC [µg/mL]		
		E	F10	F20
*Staphylococcus aureus*	*A. graveolens*	300	250	250
	*D. carota*	400	150	100
	*P. crispum*	700	200	150
*Staphylococcus* *epidermidis*	*A. graveolens*	400	250	250
	*D. carota*	100	100	50
	*P. crispum*	250	150	150
*Bacillus subtilis*	*A. graveolens*	250	nd	nd
	*D. carota*	200	250	250
	*P. crispum*	250	200	nd
*Staphylococcus capitis*	*A. graveolens*	300	250	200
	*D. carota*	250	150	50
	*P. crispum*	150	150	100
*Micrococcus luteus*	*A. graveolens*	nd	250	200
	*D. carota*	300	150	200
	*P. crispum*	300	200	200
*Yersinia enterocolitica*	*A. graveolens*	nd	nd	400
	*D. carota*	nd	300	200
	*P. crispum*	nd	nd	nd
*Pseudomonas aeruginosa*	*A. graveolens*	nd	300	200
	*D. carota*	nd	150	150
	*P. crispum*	300	200	150

nd—not detected.

## Data Availability

The data presented in this study are available on request from the corresponding author.

## Supplementary Materials

The following supporting information can be downloaded at: https://www.mdpi.com/article/10.3390/molecules30050983/s1, Figure S1. Base peak chromatograms of water extracts from *P. crispum* (red line), *A. graveolens* (blue line), *D. carota* (green line) (a) and MS and UV-Vis spectrum of the main phenolic constituent identified as Apiin (b). Table S1. MS data extracted from the main peaks found in the root extract of *P. crispum*. Table S2. MS data extracted from the main peaks found in the root extract of *A. graveolens*. Table S3. MS data extracted from the main peaks found in the root extract of *D. carota*.
