# Supplementary material for: Apiaceae Bioferments Obtained by Fermentation with Kombucha as an Important Source of Active Substances for Skin Care
Source: Molecules. 2025 Feb 20;30(5):983. doi: 10.3390/molecules30050983 (PMC11902125; doi:10.3390/molecules30050983)
Supplement: Supplementary file 1 [file molecules-30-00983-s001.zip › molecules-3481647-supplementary.pdf]

molecules

MDPI

Article

Apiaceae bioferments obtained by fermentation with kombucha as an Important Source of Active Substances for Skin Care

Zofia Nizioł-Łukaszewska [1]*, Aleksandra Ziemlewska [1], Martyna Zagórska-Dziok [1], Agnieszka Mokrzyńska [1], Magdalena Wójciak [2] and Ireneusz Sowa [2,]

[1] Department of Technology of Cosmetic and Pharmaceutical Products, Medical College, University of Information Technology and Management in Rzeszow, Sucharskiego 2, 35-225 Rzeszow, Poland
[2] Department of Analytical Chemistry, Medical University of Lublin, Aleje Raclawickie 1, 20-059 Lublin, Poland
* Correspondence: e-mail@e-mail.com; Tel.: (optional; include country code; if there are multiple corresponding authors, add author initials)

a

b

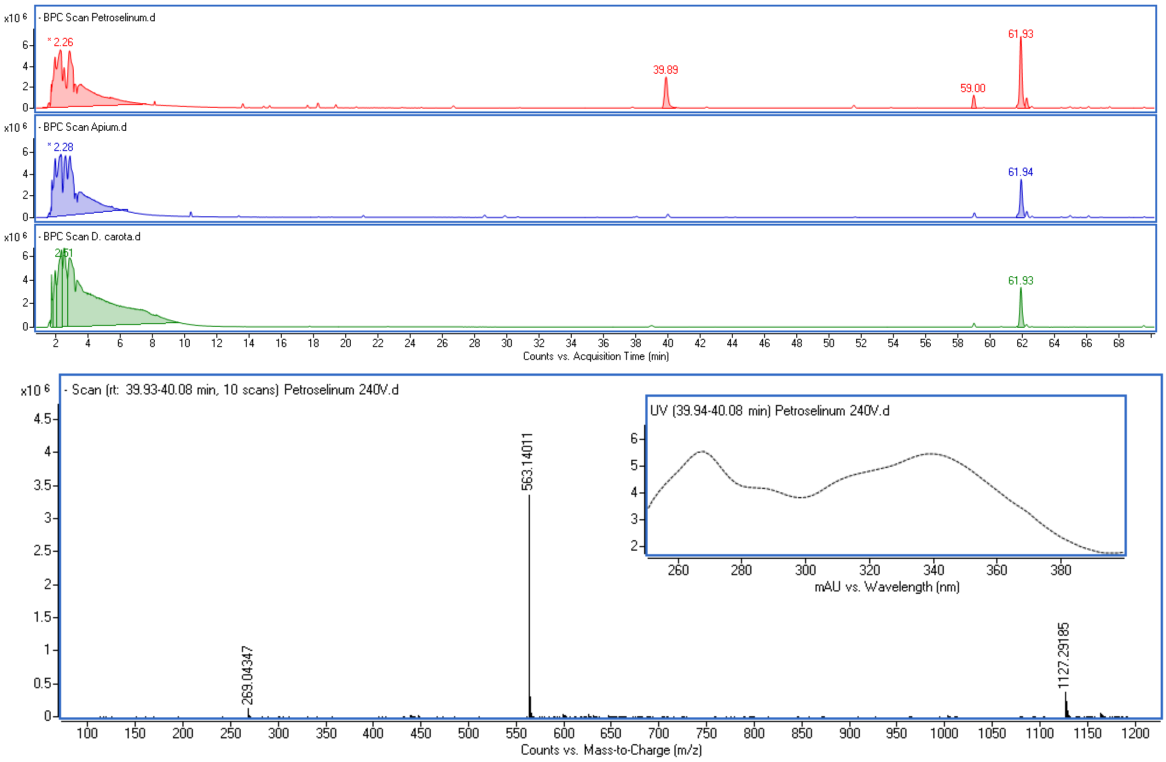

Figure S1. Base peak chromatograms of water extracts from *P. crispum* (red line), *A. graveolens* (blue line), *D. carota* (green line) (a) and MS and UV-Vis spectrum of the main phenolic constituent identified as Apiin (b).

Table S1. MS data extracted from the main peaks found in the root extract of *P. crispum*.

R_T (min.)	Mass data (m/z-H)	Formula	Δ ppm	Component
8.12	353.14559	$C_{24}H_{26}O_{10}$	0.76	unknown
13.60	445.13738	$C_{19}H_{26}O_{12}$	5.0	unknown
14.90	425.16779	$C_{17}H_{30}O_{12}$	3.14	unknown
15.27	417.10559	$C_{17}H_{22}O_{12}$	4.16	unknown
15.40	351.12549	$C_{21}H_{20}O_5$	4.81	unknown
17.62	401.14676	$C_{18}H_{26}O_{10}$	3.58	unknown
18.25	351.13167	$C_{14}H_{24}O_{10}$	5.68	unknown
19.38	381.17677	$C_{16}H_{30}O_{10}$	0.39	unknown
20.65	371.09981	$C_{16}H_{20}O_{10}$	3.87	unknown
23.52	473.16667	$C_{21}H_{30}O_{12}$	0.46	unknown
24.68	695.2199	$C_{32}H_{40}O_{17}$	0.9	unknown
26.70	579.19493	$C_{24}H_{36}O_{16}$	3.23	unknown
39.89	563.14011	$C_{26}H_{28}O_{14}$	-0.92	Apigenin-7-apioglucoside (Apiin)
40.19	431.09798	$C_{21}H_{20}O_{10}$	-0.90	Apigenin 7-glucoside
51.55	301.20232	$C_{16}H_{30}O_5$	0.9	Hydroxyhexadecanedioic acid
58.92	269.04601	$C_{15}H_{10}O_5$	1.17	Apigenin
59.0	327.21922	$C_{18}H_{32}O_5$	4.64	Trihydroxyoctadecadienoic acid
61.93	329.23488	$C_{18}H_{34}O_5$	4.64	Trihydroxyoctadecenoic acid
75.17	311.22377	$C_{18}H_{32}O_4$	3.16	Octadecenedioic acid

Table S2. MS data extracted from the main peaks found in the root extract of *A. graveolens*.

R_T (min.)	Mass data (m/z-H)	Formula	Δ ppm	Component
10.36	203.08309	$C_{11}H_{12}N_2O_2$	2.40	Tryptophan
13.35	299.07763	$C_{13}H_{16}O_8$	1.30	Hydroxybenzoic acid glucoside
18.25	351.1313	$C_{14}H_{24}O1_0$	4.63	unknown
19.14	431.19333	$C_{20}H_{32}O_{10}$	2.45	unknown
20.08	391.19896	$C_{18}H_{32}O_9$	4.09	unknown
28.62	559.16919	$C_{24}H_{32}O_{15}$	4.19	unknown
29.87	413.14685	$C_{19}H_{26}O_{10}$	3.69	unknown
30.69	589.17984	$C_{25}H_{34}O_{16}$	4.12	unknown
35.62	187.09805	$C_9H_{16}O_4$	2.49	Azelaic Acid
38.07	427.16332	$C_{20}H_{28}O_{10}$	5.49	unknown
40.02	563.14295	$C_{26}H_{28}O_{14}$	4.11	Apigenin-7-apioglucoside (Apiin)
40.25	431.09701	$C_{21}H_{20}O_{10}$	-3.15	Apigenin 7-glucoside
51.53	301.20225	$C_{16}H_{30}O_5$	0.67	Hydroxyhexadecanedioic acid
58.94	269.04559	$C_{15}H_{10}O_5$	0.16	Apigenin
59.04	327.21929	$C_{18}H_{32}O_5$	4.85	Trihydroxyoctadecadienoic acid
61.94	329.23408	$C_{18}H_{34}O_5$	2.22	Trihydroxyoctadecenoic acid
75.15	311.22337	$C_{18}H_{32}O_4$	1.88	Octadecenedioic acid

Table S3. MS data extracted from the main peaks found in the root extract of *D. carota*.

R_T (min.)	Mass data (m/z-H)	Formula	Δ ppm	Component
17.75	431.12067	$C_{18}H_{24}O_{12}$	2.71	unknown
18.50	371.09735	$C_{16}H_{20}O_{10}$	-2.74	unknown
38.81	193.05112	$C_{10}H_{10}O_4$	2.51	unknown
59.04	327.21783	$C_{18}H_{32}O_5$	0.40	Trihydroxyoctadecadienoic acid
61.93	329.23502	$C_{18}H_{34}O_5$	5.06	Trihydroxyoctadecenoic acid
